# A prioritization tool for cilia-associated genes and their *in vivo* resources unveils new avenues for ciliopathy research

**DOI:** 10.1242/dmm.052000

**Published:** 2024-10-14

**Authors:** Robert E. Van Sciver, Tamara Caspary

**Affiliations:** Department of Human Genetics, Emory University School of Medicine, Atlanta, GA 30322, USA

**Keywords:** Polycystic kidney disease, Cilia, Mouse alleles, Zebrafish mutants, Systematic phenotyping

## Abstract

Defects in ciliary signaling or mutations in proteins that localize to primary cilia lead to a class of human diseases known as ciliopathies. Approximately 10% of mammalian genes encode cilia-associated proteins, and a major gap in the cilia research field is knowing which genes to prioritize to study and finding the *in vivo* vertebrate mutant alleles and reagents available for their study. Here, we present a unified resource listing the cilia-associated human genes cross referenced to available mouse and zebrafish mutant alleles, and their associated phenotypes, as well as expression data in the kidney and functional data for vertebrate Hedgehog signaling. This resource empowers researchers to easily sort and filter genes based on their own expertise and priorities, cross reference with newly generated -omics datasets, and quickly find *in vivo* resources and phenotypes associated with a gene of interest.

## Teaser

**Summary:** By compiling data on primary cilia-associated genes, their corresponding mouse and zebrafish alleles, and phenotypes, we provide an editable, Excel-based tool to help researchers identify and prioritize genes for study.

## INTRODUCTION

The Human Genome Project aimed to revolutionize our understanding of human health and disease by revealing the function of every gene (U.S. Department of Health and Human Services and Department of Energy, 1990). Fulfilling this goal requires a list of all the genes and tools available to manipulate and interrogate each gene's function. The list of genes arrived with the first draft human genome sequence in 2001, and tools such as siRNA or RNA interference enabled any gene product to be depleted, facilitating systematic interrogation of gene function (Lander et al., 2001; Venter et al., 2001). Propelled by the promise and achievements of the Human Genome Project, subsequent international efforts aimed to generate functional null alleles of every gene in multiple models including mouse, zebrafish and yeast (Austin et al., 2004; Auwerx et al., 2004; Giaever and Nislow, 2014; Dickinson et al., 2016; Bradford et al., 2022; Giaever et al., 2002; Groza et al., 2022; Ju et al., 2022). In mouse, mutations in 18,270 protein-coding genes are currently available, covering roughly three-quarters of the known protein-coding genes in the mouse genome, and a subset of these are being systematically phenotyped (Dickinson et al., 2016; Groza et al., 2022). Similarly, coordinated efforts in zebrafish now catalog mutations in 75% of the protein-coding genes in the zebrafish genome with 19,853 mutant/morphant fish available (Bradford et al., 2022). Although some genes attracted the attention of many studies, other genes have yet to be interrogated. A key question that hinders efforts is how to prioritize which of these genes to study. Individual investigators are motivated to focus on certain genes by a myriad of factors and should continue to do so. Additionally, with the abundance of data provided today by various -omics approaches, we can now prioritize unstudied genes based on features including their cell-type expression pattern, timing of expression, subcellular localization or binding partners. We built a tool that exemplifies how such prioritization can work, focusing on genes that encode proteins associated with primary cilia.

Primary cilia are microtubule-based organelles that project from nearly every cell in the body (Pazour and Witman, 2003; Hoey et al., 2012). Once thought to be vestigial organelles, primary cilia became a focus of studies in the past two decades because they were linked to several signaling pathways important for development and disease states (Pan and Snell, 2002; Pazour et al., 2002; Yoder et al., 2002; Huangfu et al., 2003; Han et al., 2009; Wong et al., 2009; Menezes and Germino, 2009; Goetz and Anderson, 2010). Primary cilia are structurally composed of nine microtubule doublets that form the backbone of the projection, known as the axoneme. The axoneme is covered by a phospholipid bilayer that is contiguous with the cellular plasma membrane. Proteins enriched in the ciliary membrane regulate its phospholipid composition, imparting a distinct membrane fluidity and protein composition compared with that of the plasma membrane (Bae et al., 2009; Chavez et al., 2015; Garcia-Gonzalo et al., 2015). At the base of the cilium is a ciliary transition zone that controls protein traffic into and out of the cilium (Gonçalves and Pelletier, 2017; Mercey et al., 2024). Together, the transition zone and ciliary membrane create a privileged compartment allowing primary cilia to function as a fundamental cellular organelle.

There is a pressing need to know the function of cilia-associated proteins, because defects in primary cilia structure or in proteins that localize to primary cilia lead to a class of genetic disorders known as ciliopathies (Hildebrandt et al., 2011; Anvarian et al., 2019; McConnachie et al., 2021). Some ciliopathies are rare, including Bardet­­–Biedl syndrome, orofaciodigital syndrome, Joubert syndrome and Meckel syndrome, whereas others are more common, such as polycystic kidney disease and Von Hippel–Lindau disease. Ciliopathies often present with a multitude of symptoms affecting the eyes, brain, skeleton, kidneys and liver, among other organs (Reiter and Leroux, 2017). Understanding primary cilia signaling and defects that lead to ciliopathies will be crucial for developing targeted therapies. In our collective drive to understand these ciliary signaling pathways, a comprehensive understanding of the proteins that make up the specialized ciliary protein milieu is critical.

To aid in these efforts, we developed an Excel-based spreadsheet with key information that investigators can use to sort and prioritize subsets of cilia-associated genes, depending on their research interests. We first compiled an inventory of genes encoding proteins associated with primary cilia. We next examined the *in vivo* vertebrate resources available for each of these genes in mouse and zebrafish, cataloging alleles that have and have not been analyzed. Lastly, we provided gene-driven, disease-driven and process-driven examples of how we envision this tool being a useful resource for researchers. We demonstrated the utility of this tool by applying it to one of the most common monogenic diseases – polycystic kidney disease. Because primary cilia have such a large impact on kidney function, we focused on kidney expression data for each of the ciliary genes in our spreadsheet (Calvet, 2002; Dell, 2015; Ma et al., 2017; Devlin and Sayer, 2019). Vertebrate Hedgehog (Hh) signaling requires intact/functioning primary cilia and potentially influences cystic kidney phenotypes (Huangfu et al., 2003; Maezawa et al., 2012; Silva et al., 2018; Ma et al., 2019; Hsieh et al., 2022). We overlaid results from two independent Hh screens on our tool to show how ciliary signaling can inform which genes are prioritized (Breslow et al., 2018; Pusapati et al., 2018). This spreadsheet will supplement individual investigators’ intuition, facilitating prioritization of cilia-associated genes and their roles in signaling pathways, and highlighting available vertebrate resources to further their studies *in vivo*.

## RESULTS

### Compiling a ‘parts list’ for primary cilia

The first step in creating our ciliary tool was compiling an inventory of proteins that localize to or are enriched in primary cilia. Over the past two decades, researchers using discovery-based approaches like RNA sequencing (RNA-seq), siRNA screens or proximity labeling identified numerous putative ciliary proteins. Although quite successful, such a variegated approach presents challenges: some datasets provide only a list of proteins, while others link the ciliary gene to disease, and others investigate the ciliary gene's role in altering known ciliary signaling pathways. Many datasets use different cell types or tissues, and growing evidence suggests that different cell types and tissues have specialized ciliary functions and likely unique ciliary composition. We combed through existing datasets and compiled a unified database of 1999 unique ciliary genes, representing ∼10% of all known human genes (see Table S1 and Materials and Methods) (Arnaiz et al., 2009, 2014; van Dam et al., 2013, 2019; Reiter and Leroux, 2017; Vasquez et al., 2021; Mehta et al., 2022; Elliott et al., 2023). As a unified database, we overcame hurdles including the lack of unity across the databases and manually curated missing or incorrectly cross-referenced data.

### Mouse alleles and zebrafish mutants and resources, and their respective phenotypes

**Fig. 1.**
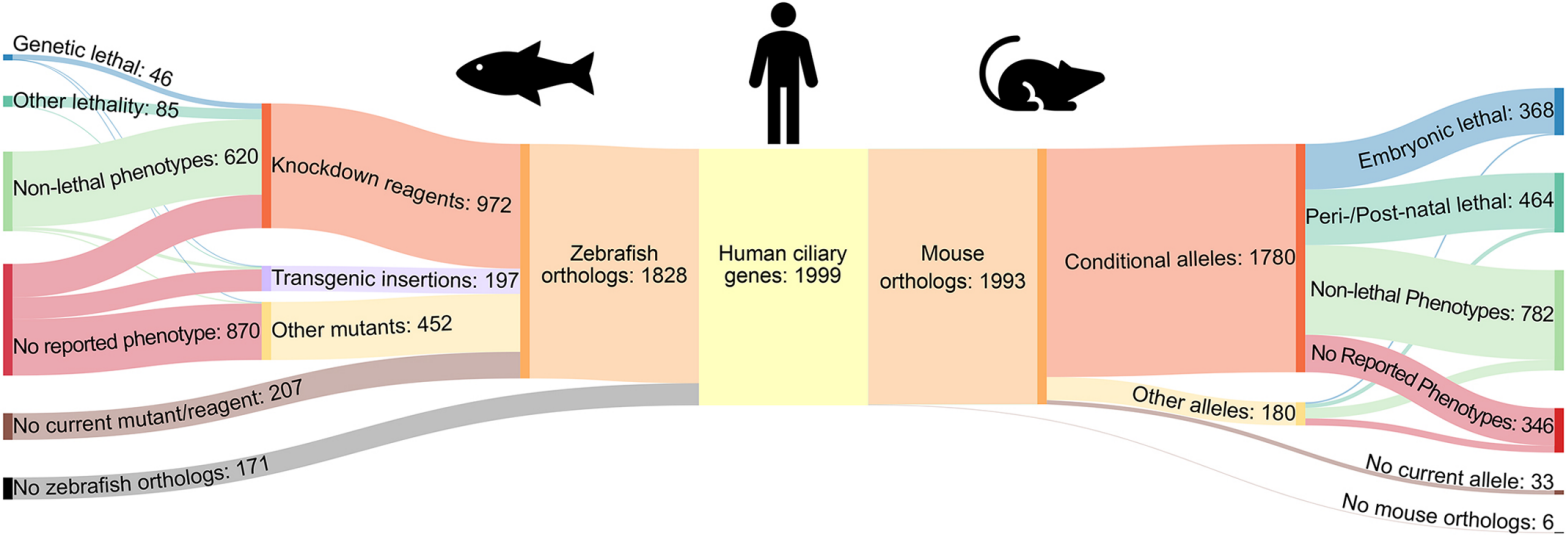
**Sankey chart of ciliary genes, resources and phenotypes in zebrafish and mouse.** Sankey plot of the 1999 compiled human ciliary genes shows the zebrafish and mouse orthologs and reagents available as well as reported lethality phenotypes, and non-lethal phenotypes across species.

We next asked which of these ciliary genes have available *in vivo* vertebrate models (Fig. 1). Mouse and zebrafish are powerful and complementary organisms for studying cilia-related phenotypes. We focused on mouse and zebrafish as vertebrate models for studying primary cilia because of the vertebrate-specific link of primary cilia with Hh signaling. One factor distinguishing zebrafish and mouse is the timing of zygotic transcription. In mice, zygotic genome activation starts at the two-cell stage, resulting in embryonic lethality for many ciliary mutants, precluding their analysis in specific tissues without the use of conditional alleles. In zebrafish, early embryo development relies on maternally expressed genes and circumvents the early lethality seen in mice with ciliary mutants, allowing for straightforward analysis of phenotypes that would require conditional analysis in mice, such as cystic kidneys. However, as mammals, mice share relevant physiology with human development and disease states. Together, both mouse and zebrafish have numerous alleles available in their respective databases and serve as complementary systems to better our understanding of the function of cilia-associated genes.

Of the 1999 identified human ciliary genes, there are mouse orthologs for 1993 (98%) (Fig. 1, right). Mouse Genome Informatics (MGI) and MouseMine indicated that 2075 mouse alleles are available for 1960 of the identified human ciliary genes (Baldarelli et al., 2024; https://www.mousemine.org). Of these, 1780 are available as conditional alleles and 180 are constitutive mutants. There are no alleles currently available for 33 of the mouse genes. We mined MGI's cataloged information on each of the genes for reported phenotypes. We categorized phenotypes as embryonic lethal, peri-/post-natal lethal or non-lethal phenotypes, or as no reported phenotypes (Fig. 1, right). Over 40% (832) of the analyzed alleles present with embryonic (368) or early post-natal (464) lethality phenotypes in mice. Of these 832 lethal phenotypes, conditional alleles are available for 789 genes, allowing researchers to bypass lethal phenotypes with spatial and temporal control. An additional 782 human ciliary genes have mouse orthologs and alleles that present with non-lethal phenotypes. Lastly, 346 mouse alleles are currently available, with no reported phenotypes. Closer examination of these alleles will likely yield cilia-associated phenotypes.

We used the Zebrafish Information Network (ZFIN) to assess the availability of fish mutants for the 1999 unique human ciliary genes (Fig. 1, left). ZFIN identified 2321 zebrafish orthologs corresponding to 1828 (91%) of the identified human ciliary genes. Of these zebrafish orthologs, 972 are available with knockdown reagents, including CRISPR, morpholinos or transcription activator-like effector nucleases (TALENs). An additional 197 ciliary genes are available as transgenic insertions, and 452 are available as other mutants (e.g. indels, point mutants, complex substitutions, etc.). In total, zebrafish mutants and resources are available for 1621 (81%) of the identified ciliary genes. Of the total identified ciliary genes, 207 have corresponding zebrafish orthologs, but no reagent or mutant available in zebrafish. Roughly half of the 1621 disrupted zebrafish genes display embryonic lethality (46), non-embryonic lethality (85) or non-lethal (620) phenotypes (Fig. 1, left). These phenotypes correspond to 751 of the human ciliary genes. The low frequency of embryonic lethality (∼2%) likely reflects the strong maternal effect during early development in fish. Finally, 870 mutants or morphants exhibit no reported phenotype.

**Fig. 2.**
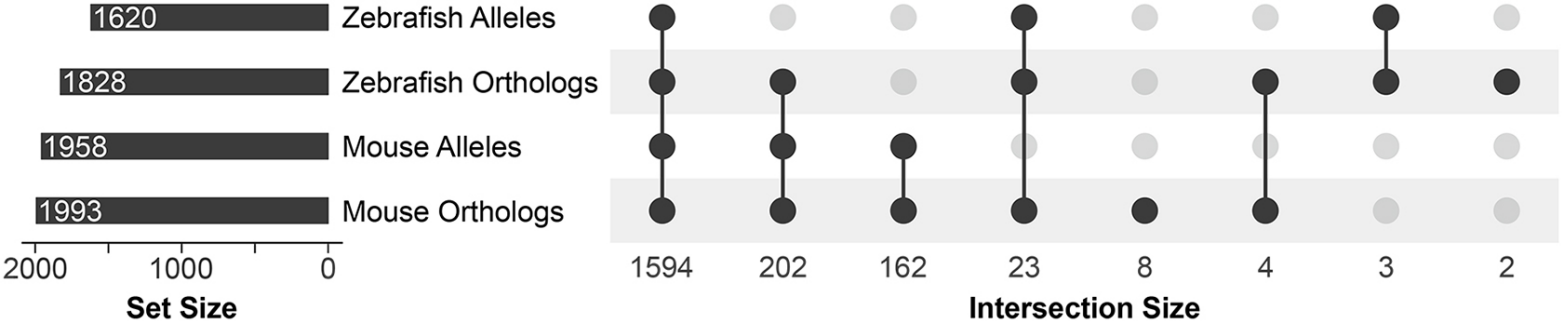
**Zebrafish and mouse orthologs and alleles of the compiled ciliary genes.** An UpSet plot showing intersections of the compiled human ciliary genes with their zebrafish and mouse orthologs and alleles.

In total, 1594 of the 1999 identified human ciliary genes have alleles and knockdown reagents in both mouse and zebrafish (Fig. 2). Mouse alleles are exclusively available for 364 of the human ciliary genes with either no corresponding ortholog (162) or with no allele (202) in zebrafish. Conversely, 26 of the human ciliary genes have resources available only in zebrafish, with no current mouse alleles. At least one ortholog in either species corresponds to every gene identified in this human cilia-associated dataset, and alleles from either model cover 1984 (99%) of the unified list of human ciliary genes. Together, mouse and zebrafish provide excellent vertebrate systems for the study of ciliary genes, covering the majority of ciliary genes meriting investigation.

### Ciliary regulators of Hh signaling

The primary cilium is crucial in vertebrate Hh signaling to regulate the balance of pathway activation and repression (Goetz and Anderson, 2010; Briscoe and Therond, 2013; Bangs and Anderson, 2017). Suppressed Hh signaling can result in developmental defects, while over-activation of Hh signaling can lead to pathologies such as medulloblastoma (Jiang and Hui, 2008). We included data from two independent high-throughput CRISPR-based Hh screens in NIH 3T3 cells, allowing a researcher to quickly determine which of the ciliary genes are positive regulators, negative regulators or attenuators of Hh signaling (in mouse fibroblast-based assays) (Breslow et al., 2018; Pusapati et al., 2018). Moreover, the researcher can also see what *in vivo* vertebrate models are available for studying these genes. Both screens identified 52 ciliary genes as positive regulators of Hh signaling. Mutant mouse alleles are available for all 52 ciliary genes identified as positive Hh regulators, with 49 available as conditional alleles. In mice, 47 of the alleles present with embryonic or peri-natal lethality, and only two mutants currently have no noted phenotypes. Similarly, zebrafish mutants or knockdown reagents are available for 41 of the ciliary genes identified as positive Hh regulators. Mutations in three positive Hh-regulating genes (*gli2a*, *cc2d2a* and *smo*) result in genetic lethality in zebrafish, and mutations in 17 positive Hh-regulating genes currently have no reported phenotypes. These functional data provide additional insights into one known signaling pathway linked with primary cilia. Because this resource is an editable spreadsheet with multiple points for cross referencing datasets, a researcher can easily customize the dataset to their interests in a similar manner.

### Kidney phenotypes and expression data

**Fig. 3.**
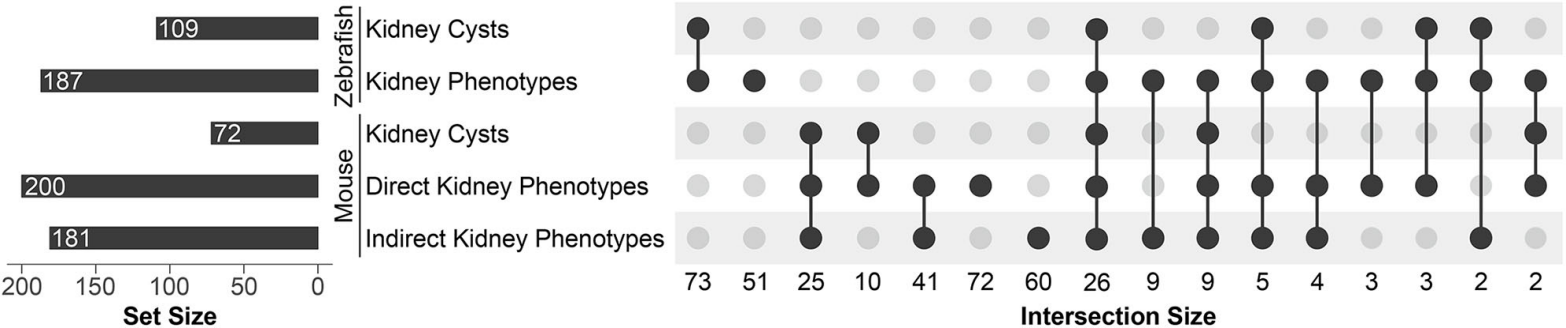
**Kidney phenotypes caused by ciliary mutants in mouse and zebrafish models.** An UpSet plot showing intersection of kidney cysts and other kidney phenotypes reported in zebrafish and mouse mutants.

The kidney is especially sensitive to phenotypes due to loss of function of cilia-associated genes, and many ciliopathies present with cystic kidney phenotypes (Devlin and Sayer, 2019; Santoni et al., 2020). We identified 594 genes in the dataset that displayed kidney-associated phenotypes in either zebrafish or mouse. We categorized these as kidney cysts or kidney phenotypes; in mouse, we subcatagorized the kidney phenotypes as being direct (e.g. enlarged kidney or abnormal kidney morphology) or indirect [e.g. altered renal function, such as abnormal blood urea nitrogen (BUN) or changes in urine content]. MGI and MouseMine reported cystic kidney phenotypes in 90 alleles, corresponding to 72 human genes (Fig. 3) (Baldarelli et al., 2024; https://www.mousemine.org). In addition, 268 alleles corresponding to 200 human genes displayed explicit kidney phenotypes, although renal cysts may not have been examined. Furthermore, we categorized an additional 96 alleles as having indirect kidney phenotypes, without any reported cysts or direct kidney phenotypes. These 96 alleles have a strong likelihood of presenting with renal phenotypes if the kidneys are examined more thoroughly.

Inclusion of zebrafish phenotypes complement what is known in mice, and together the two models cover more human genes than either system alone. ZFIN described 114 zebrafish mutants/morphants, corresponding to 109 human ciliary genes, as displaying kidney cysts. Another 78 zebrafish alleles presented with additional kidney phenotypes, although cystic kidneys were not reported explicitly. In mice, a conditional allele in kidney may lead to cystic phenotypes, whereas the null allele is embryonic lethal – precluding its study without a conditional allele. The ability of zebrafish mutants to bypass this early lethality reveals renal cystic phenotypes and interesting genes that could be worth pursuing in mice with kidney-specific Cre drivers and conditional alleles to examine their roles in cystogenesis. To this point, we identified 73 genes exclusively in zebrafish presenting with cystic kidney phenotypes, and an additional 51 genes had other kidney phenotypes. None of these genes had a similar report of cystic phenotypes nor direct/indirect kidney phenotypes in mice.

Knowing which genes are expressed in specific segments of the nephron and their relative expression pattern is key to understanding their contribution to kidney phenotypes. We overlaid two independent RNA-seq datasets onto our spreadsheet, allowing for quick analysis of expression patterns in the kidney (Ransick et al., 2019; Chen et al., 2021). The first dataset performed single-cell RNA-seq on adult mouse kidneys, whereas the second dataset performed full-length RNA-seq on microdissected mouse renal tubules. Together, these datasets reveal expression patterns of the cilia-associated genes in different segments of the mouse nephron.

### Use cases

We envision a variety of uses for this tool. Below we provide several examples that use this tool to prioritize genes in a researcher's project. First, we cover how a researcher may use the tool to find available *in vivo* resources available for a gene of interest. Second, we show how a researcher may use this tool to investigate a ciliary disease of interest. Lastly, we cover how a researcher could use this tool to investigate a specific signaling pathway.

#### Use case 1: *in vivo* resources available for ciliary genes

The most straightforward use of this tool is to identify *in vivo* mouse and zebrafish models available for studying a cilia gene simply by searching the database by gene or protein name. For example, if a researcher wants to know more about aurora kinase A (*Aurka*), they can search the database for *Aurka* or use Microsoft Excel's filter function on the gene name column and type in ‘AURKA’. This filtered view enables the researcher to see in which datasets AURKA protein was identified as ciliary (as well as those datasets that did not identify this protein). The researcher can also see that this gene was found to be an attenuator of Hh signaling with a false discovery rate of less than 20%. They can then follow a link to the International Mouse Phenotyping Consortium (IMPC) to see the current phenotypes characterized in IMPC's comprehensive International Mouse Phenotyping Resource of Standardised Screens (IMPReSS) pipeline (https://www.mousephenotype.org/impress/). The researcher can see that *Aurka* is associated with embryonic lethality in mice, but that there are conditional alleles available, providing an opportunity to investigate deletion of this gene in the researcher's tissue of choice. In addition, the ZFIN ID links to all phenotypes associated with the *aurka* gene reported in zebrafish. Should the researcher decide to pursue further investigations *in vivo*, IMPC has ordering information for mouse alleles, and ZFIN provides ordering information for zebrafish mutants as well as CRISPR, morpholino and TALEN sequences (Bradford et al., 2022; Groza et al., 2022).

**Fig. 4.**
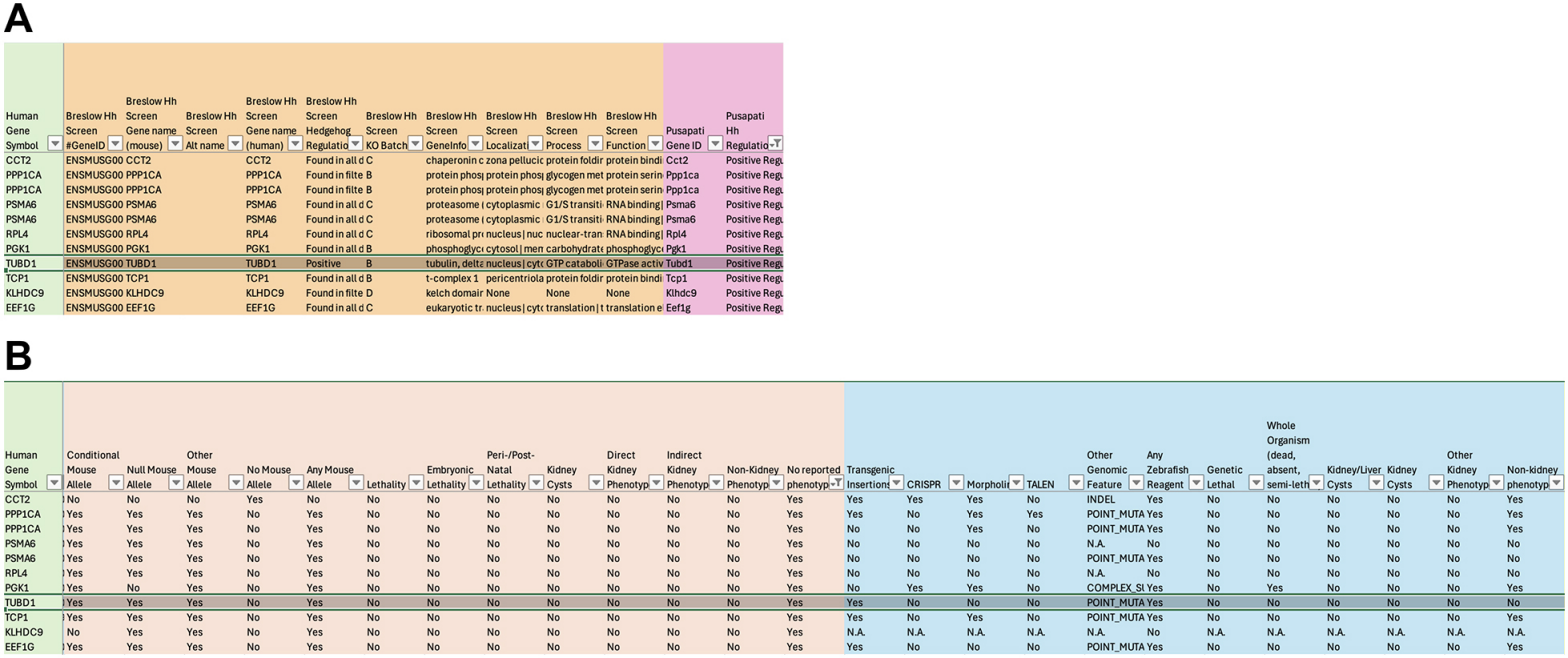
**Identifying positive Hh regulators with no reported mouse phenotype.** Screenshots of the ciliary tool. (A) Data filtered based on positive Hh regulators identified in the Pusapati et al. (2018) dataset. (B) Mouse and zebrafish alleles and reagents available for the positive Hh regulators with no reported mouse phenotype.

Another example might involve a researcher wanting to prioritize a gene among many hits in a screen. Perhaps they performed RNA-seq and want to identify the differentially expressed ciliary genes. They can compare their hits to those which are on the list of 1999 human cilia-associated genes. Inclusion of ENSEMBL IDs, HUGO Gene Nomenclature Committee (HGNC) IDs, mouse, human and zebrafish gene names in the spreadsheet facilitates cross referencing the datasets. To identify new areas of research, they might want to know which differentially expressed ciliary genes have no reported phenotypes. Filtering the dataset by ‘No reported phenotypes’ in mice provides a shortlist of follow-up candidates. They can further reduce this shortlist by filtering. For example, if they are interested in positive Hh regulators, they might select the gene *Tubd1*, which was found to be a positive regulator in both Hh screens, and pursue further investigations (Fig. 4A) (Breslow et al., 2018; Pusapati et al., 2018). This tool also shows that conditional and knockout alleles of *Tubd1* are available in mice, as are transgenic insertions and point mutants in zebrafish, to further advance their studies (Fig. 4B). Even without hits from a screen, researchers can find value in knowing that over 400 ciliary genes are listed with no noted phenotypes in mice, yet with nearly 300 conditional mouse alleles available. Filtering the dataset based on reported zebrafish phenotypes or mutant availability can help prioritize or de-prioritize genes that a researcher may wish to investigate further in mice.

#### Use case 2: prioritizing for genetic interactors in polycystic kidney disease

Another way we envision this resource to be valuable is in the study of cilia-related diseases such as polycystic kidney disease. Mouse models indicate that ciliary proteins function to inhibit a pro-cystic pathway from within cilia (Ma et al., 2013; Lee and Somlo, 2014; Ma et al., 2017). Any gene on the ciliary gene list we compiled that is also expressed in the kidney could be a candidate driver of kidney disease. Knowing which genes have and have not been analyzed for kidney phenotypes is essential for guiding future studies. A researcher can sort by known phenotypes (or lack of phenotypes) and filter their list based on those phenotypes for which ciliary genes have expression above a set threshold in renal epithelia or specific segments of the nephron. By sorting the list of ciliary genes in this tool, a researcher can refine the list and generate a shortlist of candidate genes, and immediately know what mouse and zebrafish mutant alleles are already available.

#### Use case 3: Hh signaling

**Fig. 5.**
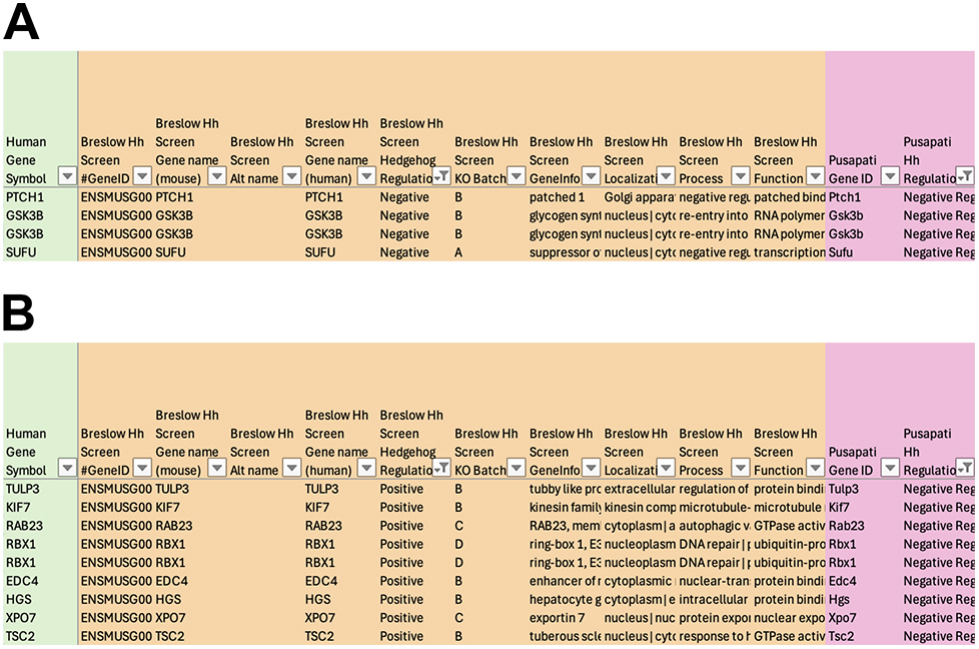
**Commonalities and differences from two independent Hh screens.** (A) List of ciliary genes identified in two independent screens as negative regulators of Hh signaling. (B) List of ciliary genes identified as positive regulators of Hh signaling in one screen and negative regulators of Hh signaling in another screen.

Further analysis of the commonalities and differences between the two independent Hh screens reveals potential areas of focus for Hh studies. Only three ciliary genes were identified in both screens as negative Hh regulators – *Ptch1*, *Sufu* and *Gsk3b* – all of which have conditional mouse mutants and zebrafish resources available (Fig. 5A). *Gsk3b* appears in this shortlist twice because zebrafish possess two orthologs, *gsk3ba* and *gsk3bb*; however, there are currently no zebrafish reagents or mutants available for the study of *gsk3bb*. Interestingly, eight genes (*Rab23*, *Tulp3*, *Kif7*, *Rbx1*, *Edc4*, *Hgs*, *Xpo7* and *Tsc2*) were identified as positive Hh regulators in one screen, but as negative Hh regulators in the other screen (Fig. 5B). All eight genes present with embryonic or perinatal lethality, and all have conditional mouse mutants and zebrafish resources available for further study. These biological processes clearly intersect, and this tool can be used as an anchoring point to identify such points of similarity and distinction among different studies.

## DISCUSSION

This compiled spreadsheet of ciliary genes serves as a resource for researchers, highlighting genetic vertebrate models available in both mice and zebrafish as well as a prototype for how to organize information in a manner that enables researchers to prioritize which genes to study. For this tool, we identified mouse alleles and zebrafish reagents for ∼98% and ∼81% of the 1999 human ciliary genes, respectively. Roughly 83% of the ciliary genes have phenotypes reported in either mouse or zebrafish, leaving 311 mouse alleles and 232 zebrafish mutants with no reported phenotype to date. With the majority of analyzed alleles presenting with notable phenotypes, the alleles with no reported phenotype are likely to present with phenotypes when carefully analyzed by experts.

Our hope is that investigators will edit and customize this tool to their research needs. In addition to mouse and zebrafish, there are many model organisms used in the study of primary and motile cilia, including *Chlamydomonas reinhardtii*, *Caenorhabditis elegans*, *Xenopus laevis* and *Drosophila melanogaster*. We chose to focus here on mouse and zebrafish models as we were motivated to concentrate on vertebrate primary cilia biology. Similar approaches could be performed analyzing orthologs in these other models. Although our resource's curation specifically focused on kidney expression and phenotypes, it is not exhaustive; other tissues or cell types of interest can be incorporated using parallel logic. For example, many ciliopathies affect the eyes and brain, so expression data from either tissue would be important for researchers interested in these tissues. Similarly, although we focus here on primary cilia, a similar approach could be applied to other organelles. The CilioGenics database provides integrated analysis of cilia-related data and contains 1996 of the 1999 cilia-associated genes we identified (Pir et al., 2024). We included the CilioGenics score and ranking, and investigators could incorporate additional data such as protein interactions or evolutionary conservation. Lastly, as additional ciliary proteins are identified, they, along with their mouse and zebrafish resources, can be manually added to this resource.

## MATERIALS AND METHODS

### Dataset acquisition

We created an all-inclusive list of ciliary genes from seven datasets: (1) SysCilia v1 (van Dam et al., 2013); (2) a list of established and candidate genes underlying ciliopathies (Reiter and Leroux, 2017); (3) CiliaCarta (van Dam et al., 2019); (4) SysCilia v2 (Vasquez et al., 2021); (5) the National Heart, Lung, and Blood Institute (NHLBI)’s primary cilium proteome database (Mehta et al., 2022); (6) the Embryonic Ciliome (Elliott et al., 2023); and (7) CilDB (Arnaiz et al., 2009, 2014). For CilDB, we narrowed our included list of genes to three conditions: (1) both human and mouse orthologs exist; (2) the number of ciliary evidences (number of studies in which the gene appears) in *Homo sapiens* is greater than or equal to three; and (3) the number of ciliary evidences in *Mus musculus* is greater than or equal to two. All databases contained a reference to the human ENSEMBL ID for each gene or protein in the dataset, except for NHLBI's primary cilium proteome database, which referenced only the mouse gene name. Many databases had significant overlap as well as distinct information for each gene. We anchored all genes using the Alliance of Genome Resources and removed duplicates, resulting in a unified database of 1999 unique ciliary genes (Bult and Sternberg, 2023; Alliance of Genome Resources Consortium, 2024). For each of the unique ciliary genes, we included the ciliogenics score and rank from the Explore data section of the CilioGenics database (https://ciliogenics.com; Pir et al., 2024).

A major gap in many of the databases centered on the lack of orthologs between species. For example, ENSEMBL does not currently list human *GAPDH* as an ortholog for mouse *Gapdh*. Therefore, any dataset that used the ENSEMBL database to identify orthologs may falsely indicate no orthologous genes found between species. To fill this gap, we manually curated missing orthologs in our database.

### Mouse alleles and phenotypes

To better understand mouse models and phenotypes corresponding to our gene list, we used the Alliance of Genome Resources (https://www.alliancegenome.org), MGI (https://www.informatics.jax.org) and MouseMine (https://www.mousemine.org). We first generated a list of orthologs using the Alliance of Genome Resources’ SimpleMine tool. We entered all of the unique HGNC IDs and used the output for mouse orthologs to obtain a list of all mouse orthologs for those genes, resulting in 2075 unique MGI numbers corresponding with 1993 HGNC IDs. We performed a batch query through MGI's website using the unique MGI numbers. For available mouse allele information, we exported the resulting batch query table to MouseMine. In MouseMine, we clicked Manage Columns and added information on Alleles, including Symbols, Primary Identifier, Allele Type and Allele Attributes. We used the resulting information from Allele Attributes to determine the availability of mouse alleles such as conditional, nulls or other mutant alleles. In MGI, we also exported mammalian phenotypes associated with each of the MGI Numbers. We queried this list based on lethality, cystic kidneys, direct or indirect kidney phenotypes, or any other noted phenotypes.

### Zebrafish reagents and phenotypes

We generated a list of zebrafish orthologs using the Alliance of Genome Resources SimpleMine tool. From this, we found that 2321 zebrafish orthologs exist for 1828 of the genes in our list. We used ZFIN to determine available fish mutants and morphants, including transgenic insertions, CRISPR, TALENs, morpholinos and other reagents (Bradford et al., 2022). We also used ZFIN to analyze phenotypes associated with the ciliary dataset. We found that 846 zebrafish orthologs have reagents available, corresponding with 751 human genes in our list.

### UpSet visualization of intersecting alleles and phenotypes

UpSet visualization was performed using UpSetR Shiny App (https://gehlenborglab.shinyapps.io/upsetr/) by inputting HGNC IDs corresponding to specific orthologs, alleles and phenotypes (Lex et al., 2014).

## Supplementary Material

10.1242/dmm.052000_sup1 Supplementary information

Table S1.

## References

[DMM052000C1] Alliance of Genome Resources Consortium (2024). Updates to the Alliance of Genome Resources central infrastructure. *Genetics* 227, iyae049. 10.1093/genetics/iyae049 38552170 PMC11075569

[DMM052000C2] Anvarian, Z., Mykytyn, K., Mukhopadhyay, S., Pedersen, L. B. and Christensen, S. T. (2019). Cellular signalling by primary cilia in development, organ function and disease. *Nat. Rev. Nephrol.* 15, 199-219. 10.1038/s41581-019-0116-9 30733609 PMC6426138

[DMM052000C3] Arnaiz, O., Malinowska, A., Klotz, C., Sperling, L., Dadlez, M., Koll, F. and Cohen, J. (2009). Cildb: a knowledgebase for centrosomes and cilia. *Database* 2009, bap022. 10.1093/database/bap022 20428338 PMC2860946

[DMM052000C4] Arnaiz, O., Cohen, J., Tassin, A.-M. and Koll, F. (2014). Remodeling Cildb, a popular database for cilia and links for ciliopathies. *Cilia* 3, 9. 10.1186/2046-2530-3-9 25422781 PMC4242763

[DMM052000C5] Austin, C. P., Battey, J. F., Bradley, A., Bucan, M., Capecchi, M., Collins, F. S., Dove, W. F., Duyk, G., Dymecki, S., Eppig, J. T. et al. (2004). The knockout mouse project. *Nat. Genet.* 36, 921-924. 10.1038/ng0904-921 15340423 PMC2716027

[DMM052000C6] Auwerx, J., Avner, P., Baldock, R., Ballabio, A., Balling, R., Barbacid, M., Berns, A., Bradley, A., Brown, S., Carmeliet, P. et al. (2004). The European dimension for the mouse genome mutagenesis program. *Nat. Genet.* 36, 925-927. 10.1038/ng0904-925 15340424 PMC2716028

[DMM052000C7] Bae, Y.-K., Kim, E., L'hernault, S. W. and Barr, M. M. (2009). The CIL-1 PI 5-phosphatase localizes TRP polycystins to cilia and activates sperm in C. elegans. *Curr. Biol.* 19, 1599-1607. 10.1016/j.cub.2009.08.045 19781942 PMC2762383

[DMM052000C8] Baldarelli, R. M., Smith, C. L., Ringwald, M., Richardson, J. E., Bult, C. J. and Group, M. G. I. (2024). Mouse Genome Informatics: an integrated knowledgebase system for the laboratory mouse. *Genetics* 227, iyae031. 10.1093/genetics/iyae031 38531069 PMC11075557

[DMM052000C9] Bangs, F. and Anderson, K. V. (2017). Primary cilia and mammalian hedgehog signaling. *Cold Spring Harb. Perspect Biol.* 9, a028175. 10.1101/cshperspect.a028175 27881449 PMC5411695

[DMM052000C10] Bradford, Y. M., Van Slyke, C. E., Ruzicka, L., Singer, A., Eagle, A., Fashena, D., Howe, D. G., Frazer, K., Martin, R., Paddock, H. et al. (2022). Zebrafish information network, the knowledgebase for Danio rerio research. *Genetics* 220, iyac016. 10.1093/genetics/iyac016 35166825 PMC8982015

[DMM052000C11] Breslow, D. K., Hoogendoorn, S., Kopp, A. R., Morgens, D. W., Vu, B. K., Kennedy, M. C., Han, K., Li, A., Hess, G. T., Bassik, M. C. et al. (2018). A CRISPR-based screen for Hedgehog signaling provides insights into ciliary function and ciliopathies. *Nat. Genet.* 50, 460-471. 10.1038/s41588-018-0054-7 29459677 PMC5862771

[DMM052000C12] Briscoe, J. and Therond, P. P. (2013). The mechanisms of Hedgehog signalling and its roles in development and disease. *Nat. Rev. Mol. Cell Biol.* 14, 416-429. 10.1038/nrm3598 23719536

[DMM052000C13] Bult, C. J. and Sternberg, P. W. (2023). The alliance of genome resources: transforming comparative genomics. *Mamm. Genome* 34, 531-544. 10.1007/s00335-023-10015-2 37666946 PMC10628019

[DMM052000C14] Calvet, J. P. (2002). Cilia in PKD--letting it all hang out. *J. Am. Soc. Nephrol.* 13, 2614-2616. 10.1681/ASN.V13102614 12239253

[DMM052000C15] Chavez, M., Ena, S., Van Sande, J., De Kerchove d'Exaerde, A., Schurmans, S. and Schiffmann, S. N. (2015). Modulation of ciliary phosphoinositide content regulates trafficking and sonic hedgehog signaling output. *Dev. Cell* 34, 338-350. 10.1016/j.devcel.2015.06.016 26190144

[DMM052000C16] Chen, L., Chou, C.-L. and Knepper, M. A. (2021). A comprehensive map of mRNAs and their isoforms across all 14 renal tubule segments of mouse. *J. Am. Soc. Nephrol.* 32, 897-912. 10.1681/ASN.2020101406 33769951 PMC8017530

[DMM052000C17] Dell, K. M. (2015). The role of cilia in the pathogenesis of cystic kidney disease. *Curr. Opin. Pediatr.* 27, 212-218. 10.1097/MOP.0000000000000187 25575298 PMC4512651

[DMM052000C18] Devlin, L. A. and Sayer, J. A. (2019). Renal ciliopathies. *Curr. Opin. Genet. Dev.* 56, 49-60. 10.1016/j.gde.2019.07.005 31419725

[DMM052000C19] Dickinson, M. E., Flenniken, A. M., Ji, X., Teboul, L., Wong, M. D., White, J. K., Meehan, T. F., Weninger, W. J., Westerberg, H., Adissu, H. et al. (2016). High-throughput discovery of novel developmental phenotypes. *Nature* 537, 508-514. 10.1038/nature19356 27626380 PMC5295821

[DMM052000C20] Elliott, K. H., Balchand, S. K., Bonatto Paese, C. L., Chang, C.-F., Yang, Y., Brown, K. M., Rasicci, D. T., He, H., Thorner, K., Chaturvedi, P. et al. (2023). Identification of a heterogeneous and dynamic ciliome during embryonic development and cell differentiation. *Development* 150, dev201237. 10.1242/dev.201237 36971348 PMC10163354

[DMM052000C21] Garcia-Gonzalo, F. R., Phua, S. C., Roberson, E. C., Garcia, G., III, Abedin, M., Schurmans, S., Inoue, T. and Reiter, J. F. (2015). Phosphoinositides regulate ciliary protein trafficking to modulate hedgehog signaling. *Dev. Cell* 34, 400-409. 10.1016/j.devcel.2015.08.001 26305592 PMC4557815

[DMM052000C22] Giaever, G. and Nislow, C. (2014). The yeast deletion collection: a decade of functional genomics. *Genetics* 197, 451-465. 10.1534/genetics.114.161620 24939991 PMC4063906

[DMM052000C23] Giaever, G., Chu, A. M., Ni, L., Connelly, C., Riles, L., Véronneau, S., Dow, S., Lucau-Danila, A., Anderson, K., André, B. et al. (2002). Functional profiling of the Saccharomyces cerevisiae genome. *Nature* 418, 387-391. 10.1038/nature00935 12140549

[DMM052000C24] Goetz, S. C. and Anderson, K. V. (2010). The primary cilium: a signalling centre during vertebrate development. *Nat. Rev. Genet.* 11, 331-344. 10.1038/nrg2774 20395968 PMC3121168

[DMM052000C25] Gonçalves, J. and Pelletier, L. (2017). The ciliary transition zone: finding the pieces and assembling the gate. *Mol. Cells* 40, 243-253. 10.14348/molcells.2017.0054 28401750 PMC5424270

[DMM052000C26] Groza, T., Gomez, F. L., Mashhadi, H. H., Muñoz-Fuentes, V., Gunes, O., Wilson, R., Cacheiro, P., Frost, A., Keskivali-Bond, P., Vardal, B. et al. (2022). The International Mouse Phenotyping Consortium: comprehensive knockout phenotyping underpinning the study of human disease. *Nucleic Acids Res.* 51, D1038-D1045. 10.1093/nar/gkac972 PMC9825559 36305825

[DMM052000C27] Han, Y. G., Kim, H. J., Dlugosz, A. A., Ellison, D. W., Gilbertson, R. J. and Alvarez-Buylla, A. (2009). Dual and opposing roles of primary cilia in medulloblastoma development. *Nat. Med.* 15, 1062-1065. 10.1038/nm.2020 19701203 PMC2771737

[DMM052000C28] Hildebrandt, F., Benzing, T. and Katsanis, N. (2011). Ciliopathies. *N. Engl. J. Med.* 364, 1533-1543. 10.1056/NEJMra1010172 21506742 PMC3640822

[DMM052000C29] Hoey, D. A., Downs, M. E. and Jacobs, C. R. (2012). The mechanics of the primary cilium: An intricate structure with complex function. *J. Biomech.* 45, 17-26. 10.1016/j.jbiomech.2011.08.008 21899847 PMC3242821

[DMM052000C30] Hsieh, C.-L., Jerman, S. J. and Sun, Z. (2022). Non-cell-autonomous activation of hedgehog signaling contributes to disease progression in a mouse model of renal cystic ciliopathy. *Hum. Mol. Genet.* 31, 4228-4240. 10.1093/hmg/ddac175 35904445 PMC9759329

[DMM052000C31] Huangfu, D., Liu, A., Rakeman, A. S., Murcia, N. S., Niswander, L. and Anderson, K. V. (2003). Hedgehog signalling in the mouse requires intraflagellar transport proteins. *Nature* 426, 83-87. 10.1038/nature02061 14603322

[DMM052000C32] Jiang, J. and Hui, C. C. (2008). Hedgehog signaling in development and cancer. *Dev. Cell* 15, 801-812. 10.1016/j.devcel.2008.11.010 19081070 PMC6443374

[DMM052000C33] Ju, C., Liang, J., Zhang, M., Zhao, J., Li, L. E., Chen, S., Zhao, J. and Gao, X. (2022). The mouse resource at national resource center for mutant mice. *Mamm. Genome* 33, 143-156. 10.1007/s00335-021-09940-x 35138443

[DMM052000C34] Lander, E. S., Linton, L. M., Birren, B., Nusbaum, C., Zody, M. C., Baldwin, J., Devon, K., Dewar, K., Doyle, M., Fitzhugh, W. et al. (2001). Initial sequencing and analysis of the human genome. *Nature* 409, 860-921. 10.1038/35057062 11237011

[DMM052000C35] Lee, S. H. and Somlo, S. (2014). Cyst growth, polycystins, and primary cilia in autosomal dominant polycystic kidney disease. *Kidney Res. Clin. Pract.* 33, 73-78. 10.1016/j.krcp.2014.05.002 26877954 PMC4714135

[DMM052000C36] Lex, A., Gehlenborg, N., Strobelt, H., Vuillemot, R. and Pfister, H. (2014). UpSet: visualization of intersecting sets. *IEEE Trans. Vis. Comput. Graph* 20, 1983-1992. 10.1109/TVCG.2014.2346248 26356912 PMC4720993

[DMM052000C37] Ma, M., Gallagher, A. R. and Somlo, S. (2017). Ciliary mechanisms of Cyst formation in polycystic kidney disease. *Cold Spring Harb. Perspect Biol.* 9, a028209. 28320755 10.1101/cshperspect.a028209 PMC5666631

[DMM052000C38] Ma, M., Tian, X., Igarashi, P., Pazour, G. J. and Somlo, S. (2013). Loss of cilia suppresses cyst growth in genetic models of autosomal dominant polycystic kidney disease. *Nat. Genet.* 45, 1004-1012. 10.1038/ng.2715 23892607 PMC3758452

[DMM052000C39] Ma, M., Legue, E., Tian, X., Somlo, S. and Liem, K. F. Jr. (2019). Cell-autonomous Hedgehog signaling is not required for cyst formation in autosomal dominant polycystic kidney disease. *J. Am. Soc. Nephrol.* 30, 2103-2111. 10.1681/ASN.2018121274 31451534 PMC6830786

[DMM052000C40] Maezawa, Y., Binnie, M., Li, C., Thorner, P., Hui, C.-C., Alman, B., Taketo, M. M. and Quaggin, S. E. (2012). A new Cre driver mouse line, Tcf21/Pod1-Cre, targets metanephric mesenchyme. *PLoS ONE* 7, e40547. 10.1371/journal.pone.0040547 22792366 PMC3391250

[DMM052000C41] Mcconnachie, D. J., Stow, J. L. and Mallett, A. J. (2021). Ciliopathies and the kidney: a review. *Am. J. Kidney Dis.* 77, 410-419. 10.1053/j.ajkd.2020.08.012 33039432

[DMM052000C42] Mehta, Y. R., Lewis, S. A., Leo, K. T., Chen, L., Park, E., Raghuram, V., Chou, C.-L., Yang, C.-R., Kikuchi, H., Khundmiri, S. et al. (2022). “ADPKD-omics”: determinants of cyclic AMP levels in renal epithelial cells. *Kidney Int.* 101, 47-62. 10.1016/j.kint.2021.10.014 34757121 PMC10671900

[DMM052000C43] Menezes, L. F. and Germino, G. G. (2009). Polycystic kidney disease, cilia, and planar polarity. *Primary Cilia* 94, 273. 10.1016/S0091-679X(08)94014-0 20362096

[DMM052000C44] Mercey, O., Mukherjee, S., Guichard, P. and Hamel, V. (2024). The molecular architecture of the ciliary transition zones. *Curr. Opin. Cell Biol.* 88, 102361. 10.1016/j.ceb.2024.102361 38648677

[DMM052000C45] Pan, J. and Snell, W. J. (2002). Kinesin-II is required for flagellar sensory transduction during fertilization in chlamydomonas. *Mol. Biol. Cell* 13, 1417-1426. 10.1091/mbc.01-11-0531 11950949 PMC102279

[DMM052000C46] Pazour, G. J. and Witman, G. B. (2003). The vertebrate primary cilium is a sensory organelle. *Curr. Opin. Cell Biol.* 15, 105-110. 10.1016/S0955-0674(02)00012-1 12517711

[DMM052000C47] Pazour, G. J., San Agustin, J. T., Follit, J. A., Rosenbaum, J. L. and Witman, G. B. (2002). Polycystin-2 localizes to kidney cilia and the ciliary level is elevated in orpk mice with polycystic kidney disease. *Curr. Biol.* 12, R378-R380. 10.1016/S0960-9822(02)00877-1 12062067

[DMM052000C48] Pir, M. S., Begar, E., Yenisert, F., Demirci, H. C., Korkmaz, M. E., Karaman, A., Tsiropoulou, S., Firat-Karalar, E. N., Blacque, O. E., Oner, S. S. et al. (2024). CilioGenics: an integrated method and database for predicting novel ciliary genes. *Nucleic Acids Res.* 52, 8127-8145. 10.1093/nar/gkae554 38989623 PMC11317154

[DMM052000C49] Pusapati, G. V., Kong, J. H., Patel, B. B., Krishnan, A., Sagner, A., Kinnebrew, M., Briscoe, J., Aravind, L. and Rohatgi, R. (2018). CRISPR Screens uncover genes that regulate target cell sensitivity to the morphogen sonic hedgehog. *Dev. Cell* 44, 113-129.e8. 10.1016/j.devcel.2017.12.003 29290584 PMC5792066

[DMM052000C50] Ransick, A., Lindström, N. O., Liu, J., Zhu, Q., Guo, J.-J., Alvarado, G. F., Kim, A. D., Black, H. G., Kim, J. and Mcmahon, A. P. (2019). Single-cell profiling reveals sex, lineage, and regional diversity in the mouse kidney. *Dev. Cell* 51, 399-413.e7. 10.1016/j.devcel.2019.10.005 31689386 PMC6948019

[DMM052000C51] Reiter, J. F. and Leroux, M. R. (2017). Genes and molecular pathways underpinning ciliopathies. *Nat. Rev. Mol. Cell Biol.* 18, 533-547. 10.1038/nrm.2017.60 28698599 PMC5851292

[DMM052000C52] Santoni, M., Piva, F., Cimadamore, A., Giulietti, M., Battelli, N., Montironi, R., Cosmai, L. and Porta, C. (2020). Exploring the spectrum of kidney ciliopathies. *Diagnostics* 10, 1099. 10.3390/diagnostics10121099 33339422 PMC7766105

[DMM052000C53] Silva, L. M., Jacobs, D. T., Allard, B. A., Fields, T. A., Sharma, M., Wallace, D. P. and Tran, P. V. (2018). Inhibition of Hedgehog signaling suppresses proliferation and microcyst formation of human autosomal dominant polycystic kidney disease cells. *Sci. Rep.* 8, 4985. 10.1038/s41598-018-23341-2 29563577 PMC5862907

[DMM052000C54] U.S. Department of Health and Human Services and U.S. Department of Energy (1990). Understanding Our Genetic Inheritance. The U.S. Human Genome Project: The First Five Years. Bethesda, MD: National Institutes of Health. NIH Publication No. 90-1590.

[DMM052000C55] van Dam, T. J. P., Wheway, G., Slaats, G. G., Huynen, M. A., Giles, R. H. and Group, S. S. (2013). The SYSCILIA gold standard (SCGSv1) of known ciliary components and its applications within a systems biology consortium. *Cilia* 2, 7. 10.1186/2046-2530-2-7 23725226 PMC3674929

[DMM052000C56] van Dam, T. J. P., Kennedy, J., Van Der Lee, R., De Vrieze, E., Wunderlich, K. A., Rix, S., Dougherty, G. W., Lambacher, N. J., Li, C., Jensen, V. L. et al. (2019). CiliaCarta: An integrated and validated compendium of ciliary genes. *PLOS ONE* 14, e0216705. 10.1371/journal.pone.0216705 31095607 PMC6522010

[DMM052000C57] Vasquez, S. S. V., Dam, J. V. and Wheway, G. (2021). An updated SYSCILIA gold standard (SCGSv2) of known ciliary genes, revealing the vast progress that has been made in the cilia research field. *Mol. Biol. Cell* 32, br13. 10.1091/mbc.E21-05-0226 34613793 PMC8694072

[DMM052000C58] Venter, J. C., Adams, M. D., Myers, E. W., Li, P. W., Mural, R. J., Sutton, G. G., Smith, H. O., Yandell, M., Evans, C. A., Holt, R. A. et al. (2001). The sequence of the human genome. *Science* 291, 1304-1351. 10.1126/science.1058040 11181995

[DMM052000C59] Wong, S. Y., Seol, A. D., So, P.-L., Ermilov, A. N., Bichakjian, C. K., Epstein, E. H., Dlugosz, A. A. and Reiter, J. F. (2009). Primary cilia can both mediate and suppress Hedgehog pathway–dependent tumorigenesis. *Nat. Med.* 15, 1055-1061. 10.1038/nm.2011 19701205 PMC2895420

[DMM052000C60] Yoder, B. K., Hou, X. and Guay-Woodford, L. M. (2002). The polycystic kidney disease proteins, polycystin-1, polycystin-2, polaris, and cystin, are co-localized in renal cilia. *J. Am. Soc. Nephrol.* 13, 2508-2516. 10.1097/01.ASN.0000029587.47950.25 12239239

