# Supplementary material for: A prioritization tool for cilia-associated genes and their in vivo resources unveils new avenues for ciliopathy research
Source: Dis Model Mech. 2024 Oct 14;17(10):dmm052000. doi: 10.1242/dmm.052000 (PMC11512102; doi:10.1242/dmm.052000)
Supplement: Supplementary information [file dmm-17-052000-s1.pdf]

A

Human Gene Symbol	Breslow Hh Screen #GeneID	Breslow Hh Screen Gene name (mouse)	Breslow Hh Screen Alt name	Breslow Hh Screen Gene name (human)	Breslow Hh Screen Hedgehog Regulatio	Breslow Hh Screen KO Batch	Breslow Hh Screen GeneInfo	Breslow Hh Screen Localizati	Breslow Hh Screen Process	Breslow Hh Screen Function	Pusapati Gene ID	Pusapati Hh Regulatio
PTCH1	ENSMUSG00	PTCH1		PTCH1	Negative	B	patched 1	Golgi appara	negative regu	patched bind	Ptch1	Negative Reg
GSK3B	ENSMUSG00	GSK3B		GSK3B	Negative	B	glycogen synt	nucleus | cyt	re-entry into	RNA polymer	Gsk3b	Negative Reg
GSK3B	ENSMUSG00	GSK3B		GSK3B	Negative	B	glycogen synt	nucleus | cyt	re-entry into	RNA polymer	Gsk3b	Negative Reg
SUFU	ENSMUSG00	SUFU		SUFU	Negative	A	suppressor o	nucleus | cyt	negative regu	transcription	Sufu	Negative Reg

B

Human Gene Symbol	Breslow Hh Screen #GeneID	Breslow Hh Screen Gene name (mouse)	Breslow Hh Screen Alt name	Breslow Hh Screen Gene name (human)	Breslow Hh Screen Hedgehog Regulatio	Breslow Hh Screen KO Batch	Breslow Hh Screen GeneInfo	Breslow Hh Screen Localizati	Breslow Hh Screen Process	Breslow Hh Screen Function	Pusapati Gene ID	Pusapati Hh Regulatio
TULP3	ENSMUSG00	TULP3		TULP3	Positive	B	tubby like pro	extracellular	regulation of	protein bindi	Tulp3	Negative Reg
KIF7	ENSMUSG00	KIF7		KIF7	Positive	B	kinesin family	kinesin comp	microtubule-	microtubule	Kif7	Negative Reg
RAB23	ENSMUSG00	RAB23		RAB23	Positive	C	RAB23, mem	cytoplasm | a	autophagic v	GTPase activ	Rab23	Negative Reg
RBX1	ENSMUSG00	RBX1		RBX1	Positive	D	ring-box 1, E3	nucleoplasm	DNA repair |	ubiquitin-pro	Rbx1	Negative Reg
RBX1	ENSMUSG00	RBX1		RBX1	Positive	D	ring-box 1, E3	nucleoplasm	DNA repair |	ubiquitin-pro	Rbx1	Negative Reg
EDC4	ENSMUSG00	EDC4		EDC4	Positive	B	enhancer of r	cytoplasmic	nuclear-tran	protein bindi	Edc4	Negative Reg
HGS	ENSMUSG00	HGS		HGS	Positive	B	hepatocyte g	cytoplasm | e	intracellular	protein bindi	Hgs	Negative Reg
XPO7	ENSMUSG00	XPO7		XPO7	Positive	C	exportin 7	nucleus | nuc	protein expo	nuclear expo	Xpo7	Negative Reg
TSC2	ENSMUSG00	TSC2		TSC2	Positive	B	tuberous scle	nucleus | cyt	response to h	GTPase activ	Tsc2	Negative Reg

Fig. S1. Commonalities and differences from two independent hedgehog screens. (A) List of ciliary genes identified in two independent screens as negative regulators of Hh signaling. (B) List of ciliary genes identified as positive regulators of Hh signaling in one screen and negative regulators of Hh signaling in another screen.

Table S1.

Available for download at
https://journals.biologists.com/dmm/article-lookup/doi/10.1242/dmm.052000#supplementary-data

Disease Models & Mechanisms • Supplementary information
